# Mental health of public safety personnel: Developing a model of operational, organizational, and personal factors in public safety organizations

**DOI:** 10.3389/fpubh.2023.1140983

**Published:** 2023-03-02

**Authors:** Megan Edgelow, Agnieszka Fecica, Caroline Kohlen, Kirandeep Tandal

**Affiliations:** School of Rehabilitation Therapy, Queen's University, Kingston, ON, Canada

**Keywords:** occupational health, public safety, organizational factors, mental health, public safety personnel

## Abstract

The work of public safety personnel (PSP) such as police officers, firefighters, correctional officers, and paramedics, as well as other PSP, makes them vulnerable to psychological injuries, which can have profound impacts on their families and the communities they serve. A multitude of complex operational, organizational, and personal factors contribute to the mental health of PSP; however, to date the approach of the research community has been largely to explore the impacts of these factors separately or within single PSP professions. To date, PSP employers have predominantly focused on addressing the personal aspects of PSP mental health through resiliency and stress management interventions. However, the increasing number of psychological injuries among PSPs and the compounding stressors of the COVID-19 pandemic demonstrate a need for a new approach to the study of PSP mental health. The following paper discusses the importance of adopting a broader conceptual approach to the study of PSP mental health and proposes a novel model that highlights the need to consider the combined impacts of operational, organizational, and personal factors on PSP mental health. The **TR**i-**O**perational-**O**rganizational-**P**ersonal Factor Model (TROOP) depicts these key factors as three large pieces of a larger puzzle that is PSP mental health. The TROOP gives working language for public safety organizations, leaders, and researchers to broadly consider the mental health impacts of public safety work.

## 1. Introduction

Public safety personnel (PSP) such as police officers, firefighters, correctional officers, and paramedics, as well as border service officers, rescue personnel, operational intelligence personal, and communications operators/dispatchers work to protect the public (1–9). Individuals working in these careers have greater exposure to psychological trauma than civilians, making their mental wellness particularly relevant (10, 11). Research has revealed that PSP work is associated with higher rates of several mental health conditions including posttraumatic stress disorder (PTSD), anxiety disorders, depression, and substance use disorder as well as increased suicidal ideation, stress, and burnout compared to the general public (10). The mental wellness of PSP workers has significant ripple effects for PSP families and their communities (11).

In 2020, the Government of Canada released a national strategy on PTSD, which is heavily focused on public safety populations, making this a particularly pertinent time for research addressing PSP mental health. In addition, the unprecedented pressures of the COVID-19 pandemic have compounded the occupational stressors faced by PSPs while also highlighting the need for work addressing the mental health needs of this particular population of workers (5). Each individual's mental health is the result of a multitude of factors with the most commonly studied factors falling into the categories of operational, organizational, and personal factors.

### 1.1. Operational factors

Operational factors refer to the content of the work and include demands unique to the job and the specific pressures facing PSPs. For instance, responding to violent situations, feeling fearful of potential injury, or experiencing negative interactions with the public while on duty (12) could all be considered operational factors. Other operational factors include workload and threats to safety and risk of injury or death (12). The COVID-19 pandemic has further revealed the potential negative mental health impact of increased operational risk when performing public safety work (5).

### 1.2. Organizational factors

Organizations that employ PSPs can also contribute to work-related stress and cause negative mental health outcomes (13). Organizational factors include elements of the employment context that impact the mental health of PSPs during their work. These factors are often controlled or highly influenced by the employer and can either contribute to work related stress or act as facilitators to improve mental health outcomes, as well as job satisfaction and work efficiency (13, 14). Inadequate supervisor support or poor workplace culture, for example, can act as barriers to positive mental health outcomes (14–16).

### 1.3. Personal factors

Personal factors are unique to the individual PSP and depend on the circumstances of each person, including their family and social relationships, their overall health status, and their individual interests and activities outside of work. Personal factors can exacerbate stressors already present at work, such as poor familial support or experiencing a mental health condition (16). Personal factors can also interact with the demands of the job (i.e., operational factors) and act as either facilitators or stressors.

### 1.4. Need for more research

To date, most research has adopted a reductive strategy to the study of PSP mental health by exploring the mental health impacts of operational, organizational, and/or personal factors separately or within a single PSP profession [e.g., (4, 12, 14–20)]. However, at any one time, an individual's mental health is influenced by a variety of factors with the balance of impacts changing with different pressures. Consider, for instance, the impact of certain operational factors such as witnessing the traumatic injury of a child when also dealing with the illness of a loved one at home and knowing that organizational demands are so great that you will not be able to take any time off to process the traumatic experience or care for your loved one. Given the dynamic and complex contributions of operational, organizational, and personal factors on the mental health of a population already at an elevated risk of serious psychological injuries, it is important to move beyond the investigation of individual impacts and focus on the interplay between all three factors when exploring and addressing the mental health needs of all PSP.

The need to approach PSP mental health from a broader perspective, one that moves beyond a focus on a single category of factors, was further highlighted by the findings of a recent scoping review that explored the role that operational, organizational, and personal factors play in the mental health of PSPs (2). The review revealed that although organizational factors are most amenable to change, it is also vital to consider the impact of operational and personal factors when addressing the mental health needs of PSP (2). The intent of this paper is to propose a visual model that can serve as a foundation for examining the dynamic and often interconnected roles that operational, organizational, and personal factors play in the mental health of PSPs. The purpose of this model is also to serve not only as a roadmap for future studies and interventions focusing on PSP mental health, which could lead to amendments and further development of the model, but also a guide to help PSP organizations identify factors that can be addressed within their own context to facilitate positive changes for workplace mental health. The aim of this model is not to provide a comprehensive overview of PSP mental health, as many aspects of PSP mental health have yet to be studied, but rather to draw attention to the need to broaden our focus as researchers and employers to better address the needs of a vulnerable population that serves all of our communities.

## 2. A tri-factor model of public safety personnel mental health

**Figure 1:**
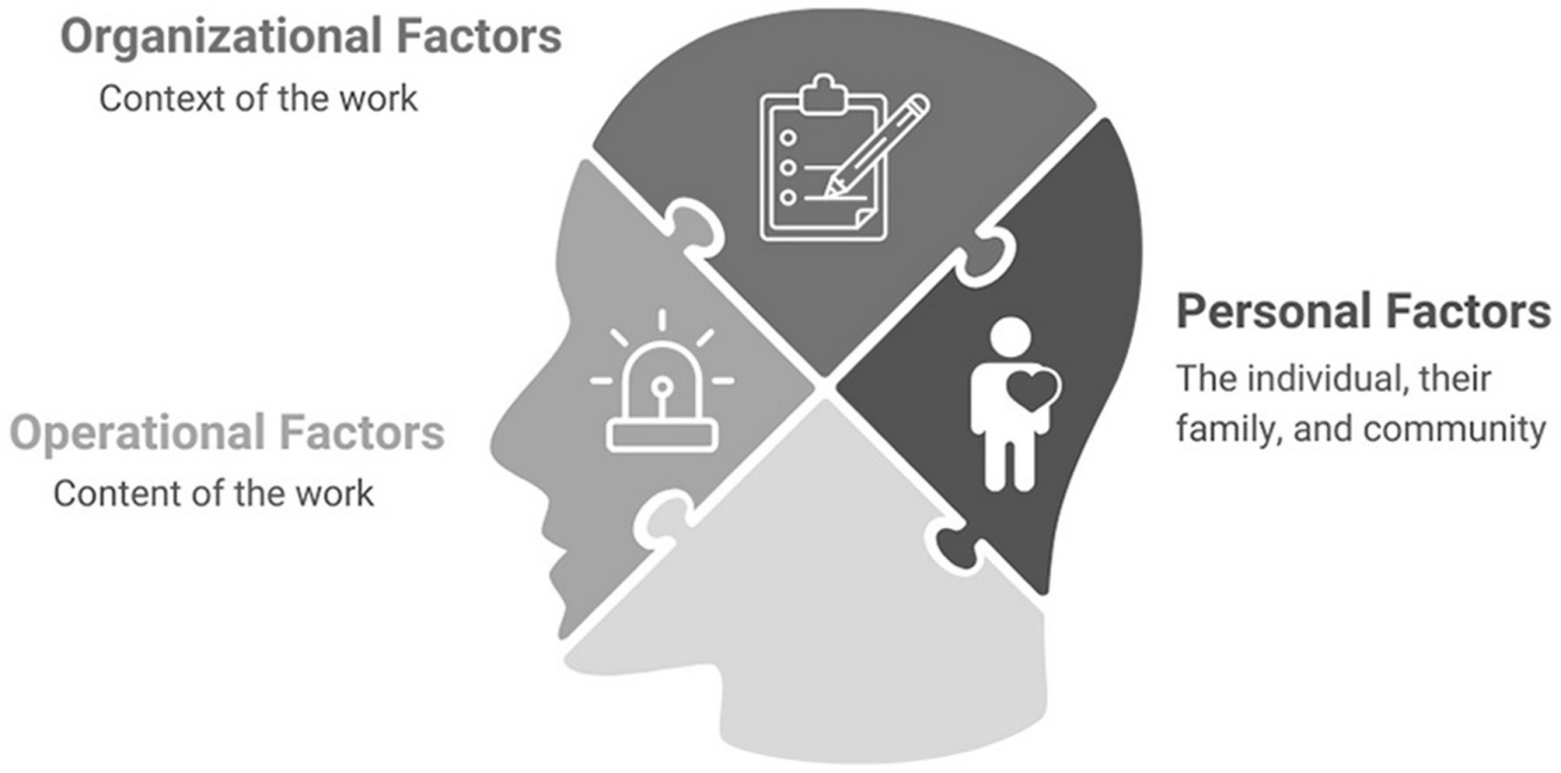
**TR**i-**O**perational-**O**rganizational-**P**ersonal Factor Model (TROOP).

This new tri-factor model focuses on the impact of three broad factors (operational, organizational, and personal) known to impact PSP mental health. The factors are depicted as three large pieces of a larger puzzle that is PSP mental health (Figure 1). In the figure, the bottom puzzle piece is left blank to symbolize the larger context, which could include external factors beyond the workplace, home, or community, as well as elements that research may not yet have focused on. No single piece is more or less important than the other; however, the salience of each can vary at any given point in time depending on the individual circumstances of PSPs.

Named the **TR**i-**O**perational-**O**rganizational-**P**ersonal Factor Model (TROOP), this tri-factor model gives visibility to these key factors as well as working language for public safety organizations, leaders, and researchers to broadly consider the mental health impacts of public safety work.

## 3. Discussion

Each one of the three factors in the TROOP include a complex network of factors that previous research has found to have an impact on PSP mental health. Using the findings from Edgelow et al.'s scoping review (2), we have created Table 1 to summarize the most common operational, organizational, and personal factors known to impact PSP mental health positively and negatively. This table also includes a representation of the amount of published scholarly work that has, to date, focused on a given factor known to impact PSP mental health. It is important to note that we are just beginning to understand which factors positively or negatively impact PSP mental health and as research progresses, more factors can be added to the table. What the model and tables highlight is that a great deal more work is required to explore the complex interplay between operational, organizational, and personal factors and their joint impacts on PSP mental health (1, 2).

**Table 1:** Summary of the top 10 factors known to impact PSP mental health [as in Edgelow et al. (2)].

**Negative operational factors**	**# of studies**	**Negative organizational factors**	**# of studies**	**Negative personal factors**	**# of studies**	**Total negative factors**
Exposure to critical incidents	21	Lack of supervisor support	23	Health conditions (mental)	26	
High workload	20	Negative workplace environment	21	Work/life/family conflict	19	
Threats or risk of violence	13	Lack of co-worker support	14	Gender	12	
Administrative duties	12	Limited resources to perform work	14	Job satisfaction	12	
Negative public interactions	12	Interpersonal conflict with colleagues	13	Poor sleep	12	
Workplace stress	12	Stigma/barriers to seeking help	13	Lack of coping skills	10	
Risk of injury	12	Leadership issues	12	Fatigue	9	
Experiencing violence	9	Overtime hours	12	Health conditions (physical)	8	
Work overload	8	Understaffing	12	Substance misuse	8	
Risk of death	5	Shift work	11	Burnout	7	
*Total*	*124*	*Total*	*145*	*Total*	*123*	***392***
**Positive operational factors**	**# of studies**	**Positive organizational factors**	**# of studies**	**Positive personal factors**	**# of studies**	**Total positive factors**
Role	2	Co-worker support	10	Job satisfaction or meaning	8	
Tenure	1	Supervisor support	8	Family support	8	
Rank	1	Autonomy	4	Gender	4	
Department setting	1	Positive workplace culture	3	Work/life/family balance	4	
Sense of safety	1	Adequate training	3	Adequate sleep	3	
		Access to mental health specialists	2	Positive coping skills	3	
		Positive leadership	2	Good physical health	3	
		Recognition of good work	2	Race	3	
		Role clarity	2	Resilience	3	
		Team dynamics	2	Social support	3	
*Total*	*6*	*Total*	*38*	*Total*	*42*	***86***
*Total operational factors*	*130*	*Total organizational factors*	*183*	*Total personal factors*	*165*	***TOTAL***
						***478***

### 3.1. Operational factors known to impact PSP mental health

Operational factors are unavoidable aspects of public safety work. In the Edgelow et al. review (2), factors known to have a positive impact on PSP mental health include work role. For example, police officers working in “operational support” roles (e.g., firearms officers, family liaison, and negotiators) roles had lower odds of developing mental health conditions compared to “investigations officers” (e.g., public protection, counter terrorism, and forensics) (17). Working in suburban, urban, and mixed departments has been associated with a lower risk to mental health compared to rural departments (21). Conversely, exposure to critical incidents (18–20) is a frequently cited negative operational factor as is high workload (22–24). Other operational factors associated with negative impacts on PSP mental health include risk of violence (25–27) and negative interactions with the public (28–30). Longer tenure and higher rank have also been negatively associated with PSP mental health (20, 21). Table 1 depicts the most well-documented factors known to impact PSP mental health positively and negatively.

### 3.2. Organizational factors known to impact PSP mental health

Organizational factors include the context in which public safety work occurs. These organizational factors have the potential to either create added stress or facilitate positive outcomes for the PSP. For instance, support from supervisors (31–33) and co-workers (34–36) can lead to higher job satisfaction and improve mental health, or conversely, a lack of support can contribute to the opposite impacts (12, 37, 38). Other negative factors include negative workplace culture (20, 39, 40), limited resources to perform the work (23, 41, 42), and work-related interpersonal conflict with colleagues (43–45) (Table 1).

### 3.3. Personal factors known to impact PSP mental health

Personal factors are unique to each individual PSP and exist outside of the work context but may interact with it. Edgelow et al. (2) found that family support (25, 46, 47) and job satisfaction (18, 48, 49) most positively impacted PSP mental health. For example, family relationships have been found to have a protective role in preventing correctional officers from attempting suicide (50). Other personal factors known to positively impact PSP mental health include work, life, and family balance (35, 51), good physical health (52, 53), and social support (46, 54). On the other hand, the most common personal factor that worsened work related stress was experiencing a mental health issue (55–57) with PTSD, anxiety, and depression being the most frequently listed diagnoses. Dealing with work, life, and family conflicts also had a negative impact on PSP mental health (47, 55, 58). When considering mental health impacts, it should be noted that there is a bi-directional relationship, in that each factor can cause increased stress on the other. Research also indicates that public safety careers negatively impacted PSPs' social life outside of work (59) due to their “unsociable” working hours and limited availability outside of work (60) (Table 1).

### 3.4. Use of the TROOP and the factors summary table

Factors related to PSP mental health have been grouped into three broad categories: operational, organizational, and personal factors. Each broad category includes several factors known to impact PSP mental health positively or negatively. The goal of this paper was to introduce the TROOP and also provide a summary of existing research on the positive and negative factors that fall within each of these three categories. Table 1 depicts the relative frequency of published scientific work focusing on a given factor (2).

Given the inherent stressors associated with PSP work and PSPs' increased risk of psychological injury, it is possible to use the TROOP (Figure 1) and Table 1 to consider how factors can be attended to within a workplace. Operational risks associated with PSP work are often thought of as inherent to the job, but all jobs with safety risks can be approached with an occupational and public health lens to reduce work-related risk. Personal factors are also not easily modified, but organizations can offer mental health supports to employees and their families and adopt policies that encourage work-life balance. Organizational factors may be the most modifiable. A recent review (1) considered the impact of work stressors on PSPs and found that organizational factors such as supervisor support, leadership styles, shift work models, staffing levels, stigma, and workplace culture are amenable to change within PSP organizations. Using the TROOP (Figure 1) can ensure that organizations consider operational, organizational, and personal factors more holistically in their efforts to improve workplace mental health.

## 4. Conclusion

This paper has proposed that researchers and employers broaden their focus with respect to PSP mental health and offers the **TR**i-**O**perational-**O**rganizational-**P**ersonal Factor Model (TROOP), a model of operational, organizational, and personal factors as a roadmap to explore and address the mental health needs of PSP. The aim of this paper was to provide a model to synthesize and depict the wide breadth of scientific work exploring factors that impact PSP mental health. In addition, the tri-factor table depicts the relative frequency of published scientific work focusing on a given factor (Table 1) (2). The overall aim of this work is to draw attention to the need to broaden our approach to future research exploring the complex factors that impact PSP mental health and to assist public safety organizations in attending to a variety of factors that impact the mental health of PSP within their organizations and our communities.

## Data availability statement

The original contributions presented in the study are included in the article/supplementary material, further inquiries can be directed to the corresponding author.

## Author contributions

ME and AF contributed to the model conception and design. All authors shared the writing of the manuscript and read and approved the final manuscript.
